# Electron-beam lithography for polymer bioMEMS with submicron features

**DOI:** 10.1038/micronano.2016.53

**Published:** 2016-11-07

**Authors:** Kee Scholten, Ellis Meng

**Affiliations:** 1 Department of Biomedical Engineering, University of Southern California, Los Angeles, CA 90089-1111, USA

**Keywords:** electron beam lithography, flexible electronics, parylene C, polymer MEMS

## Abstract

We present a method for submicron fabrication of flexible, thin-film structures fully encapsulated in biocompatible polymer poly(chloro-p-xylylene) (Parylene C) that improves feature size and resolution by an order of magnitude compared with prior work. We achieved critical dimensions as small as 250 nm by adapting electron beam lithography for use on vapor deposited Parylene-coated substrates and fabricated encapsulated metal structures, including conducting traces, serpentine resistors, and nano-patterned electrodes. Structures were probed electrically and mechanically demonstrating robust performance even under flexion or torsion. The developed fabrication process for electron beam lithography on Parylene-coated substrates and characterization of the resulting structures are presented in addition to a discussion of the challenges of applying electron beam lithography to polymers. As an application of the technique, a Parylene-based neural probe prototype was fabricated with 32 recording sites patterned along a 2 mm long shank, an electrode density surpassing any prior polymer probe.

## Introduction

Standard microfabrication techniques have been successfully deployed on polymer films including polydimethylsiloxane (PDMS), polyimide, and poly(chloro-p-xylylene) (Parylene C), yielding novel microtechnologies that exploit polymeric flexibility, optical transparency, and chemical resistance^1^. Many conform to a common archetype: surface micromachined electrical or fluidic structures encapsulated in a thin-film polymer, which acts as both the supporting substrate and the insulating layer. This approach has been adopted for the production of flexible electronics^2^, sensors^3–5^, and displays^6,7^, with considerable interest in developing biomedical microdevices for *in vivo* implantation. The combination of low hardness and low rigidity, characteristic to polymer devices, mitigates risk of tissue damage and subsequent immune response^8^, whereas the biocompatibility afforded by several inert polymers conveys negligible cytotoxicity. Examples of implantable polymer microdevices include neural probes^9–11^, cochlear implants, biomedical sensors^12,13^, and drug dispensing devices^14,15^; some, such as retinal electrodes^16,17^ and peripheral nerve interfaces^18,19^, exploit polymeric flexibility to achieve non-planar geometries. Parylene C in particular has found widespread adoption, owing to its low moisture permeability, high electrical resistivity, and designation as a USP (United States Pharmacopeia) Class VI polymer^20^.

Fabrication of polymer-based microdevices is not without obstacles. Processing is often constrained by limited thermal and radiation tolerances. Oxidation, cracking, or bubbling can occur due to high heat or irradiance exposure during lithography steps standard in semiconductor or MEMS processing. Thermal stress, thermal expansion, or damage to the polymer chains themselves are all serious concerns that preclude several high-energy fabrication procedures, and deposition methods often produce polymer films that are insufficiently planar for high-resolution patterning. As a result, polymeric devices remain constrained by limited feature resolution offered by conventional ultraviolet (UV) lithography and high-resolution structures such as those common to processes on silicon cannot be easily replicated using existing polymer micromachining techniques. This has greatly impeded the advance of polymeric biomedical implants, despite the advantages in material properties and biocompatibility. For example, although silicon-based neural probes have been developed with electrode densities of 188 sites per 4 mm probe length (245 recording sites per mm^2^)^21^, the highest reported for an analogous polymer probe is 16 sites per 1.5 mm (60 recording sites per mm^2^)^9^, a limitation of the minimum pitch and spacing of electrical connections. There has been limited work transitioning available submicron processing to flexible polymers, including few efforts exploring the integrity of submicron electrical structures when subjected to mechanical distortions inherent to flexible devices^22^.

Alternative methods for high-resolution patterning of soft polymers have found some success, and include nano-imprint lithography or stamping^23,24^, and self-assembly^1,25^; these methods allow for highly parallelized fabrication of submicron structures on polymer substrates. All such approaches pose problems for fabrication of implantable microdevices, including difficulties aligning multi-layer patterns, restrictions on material choice, and for nano-imprint lithography, use of high temperatures and pressures, which can deform polymer substrates. There are reports of patterning repeated arrays of photonic structures on Parylene C with such methods^26,27^, proving the feasibility of batch-scale fabrication on the biocompatible polymer; however, there are no such reports on the application to microdevices. For the engineer constrained by limited resolution on polymeric devices, there remains a dearth of tools and information, and these highly parallel methods are not amenable to the rapid iterative experimentation needed for device development. Electron beam lithography (EBL) provides the requisite high pattern resolution, alignment accuracy, and design freedom, but employing the method on polymer substrates is non-trivial. Insulating surfaces induce unwanted charging effects during EBL exposure, while both the beam itself and the processing required to construct EBL resist masks may damage the polymer film. Although daunting, these obstacles can be circumvented, and application of EBL on a polymer substrates such as polyethylene terephthalate has been documented, using a combination of a conductive metal coating, and careful calibration of beam energy and exposure dosage^28^. By adopting a similar approach, we seek to transfer EBL patterns to metal structures embedded in free-film polymers, for fabricating functional polymer MEMS with submicron components.

Here we present a method for fabrication of thin-film structures on flexible Parylene C with resolution approaching the nanoscale. This work was motivated by the need for greatly improved pattern resolution in the pursuit of high density, low footprint, implantable polymers MEMS. In this work, a modified EBL process was developed for application to polymer substrates, and demonstrated through the production of thin-film titanium structures with critical features as small as 250 nm on Parylene C. Parylene C was selected for study owing to the extraordinary uniformity of vapor deposited films, and its proven success in biomedical applications; titanium was selected owing to its strong adhesion to Parylene C and ubiquity among implantable devices. We describe characterization of the EBL process and critical obstacles encountered as a resource to other researchers. Submicron structures, including conducting traces, serpentine resistors, and nano-patterned electrodes, were machined and are presented as components in robust, flexible, free-film devices. The electrical and mechanical properties of the resulting structures are discussed briefly, as is the conformality of Parylene encapsulation at this size scale. Finally, we present a prototype high-density microelectrode neural probe fabricated using this approach, with electrode density greater than any prior example of a polymer-based neural probe. The devices and structures presented here represent an order-of-magnitude improvement in pattern resolution for polymer MEMS implants, and for Parylene C devices specifically. We conclude by discussing potential applications for improved and novel devices that exploit this new size regime.

## Materials and methods

### Materials and equipment

Clean silicon dies coated in a layer of Parylene C (10 μm thick) served as the base for all subsequent experiments and fabrication. Parylene coatings were created using a room temperature CVD process with a commercial coating system (PDS 2010 labcoater, Specialty Coating Systems, Indianapolis, IN, USA). EBL patterning was performed using a Raith-150 e-beam writer (Raith Nanofabrication, Dortmund, Germany), and poly(methyl methacrylate) (PMMA, 495kA6 and 950kA2) EBL resist obtained from MicroChem Corp. (Westborough, MA, USA). All fabrication and testing was performed with in-house facilities at the University of Southern California. Scanning electron microscopy (SEM) was performed at the USC Center for Electron Microscopy and Microanalysis, using a <10 nm layer of sputtered platinum to avoid charging on Parylene surfaces.

### Electron beam lithography on parylene

**Figure 1:**
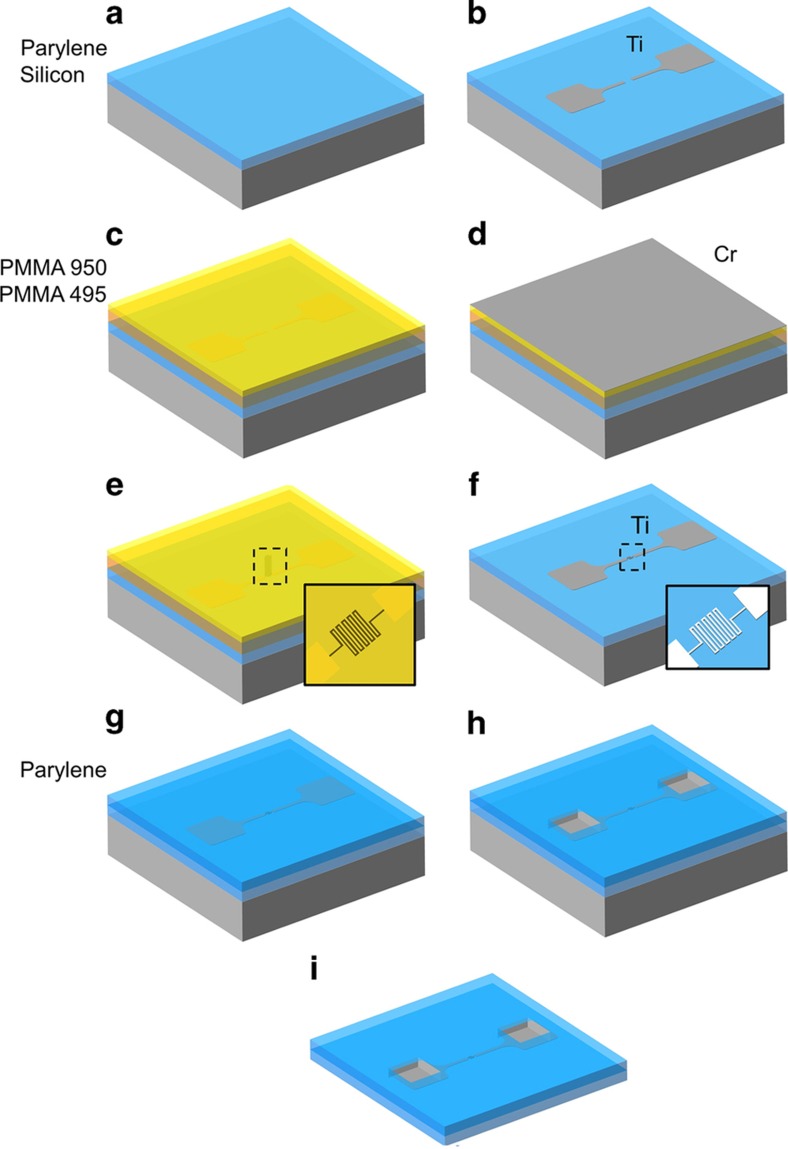
Process flow for submicron fabrication on Parylene C: (**a**) Parylene C layer 10 μm thick is deposited on a silicon carrier through chemical vapor deposition; (**b**) larger structures, such as electrodes or contact pads, are fabricated with UV lithography, metal vaporization, and liftoff; (**c**) a PMMA resist bilayer 570 nm thick is prepared with spin-coating and baked at 120 °C for 20 min; (**d**) a chromium conducting layer is deposited—15 nm thick, and the pattern is exposed with electron beam lithography; (**e**) the chromium layer is removed and the resist developed; (**f**) the submicron pattern is metalized in 150 nm of titanium using metal evaporation and liftoff; (**g**) a second Parylene C layer of 10 μm thick is deposited; (**h**) Parylene insulation above contact pads is etched with deep reactive ion etching; (i) the completed device is removed from the silicon carrier. Parylene C, poly(chloro-p-xylylene); PMMA, poly(methyl methacrylate); UV, ultraviolet.

EBL patterning was performed on Parylene C coated Si dies with and without pre-patterned features. The method is briefly illustrated in Figures 1c–f; greater details are provided in the Supplementary Information. A PMMA bilayer was selected as the e-beam resist to facilitate metallization of patterned features using liftoff. PMMA 495k (6% solids in anisole) was spun to 400 nm thickness and baked on a 120 °C hotplate for 20 min to remove residual solvent, followed by PMMA 950k (2% solids in anisole) spun to 170 nm thickness and baked at 120 °C for an additional 20 min. These bake temperatures are considerably lower than those suggested by the manufacturer, who recommend short bakes at 170–180 °C (Ref. 29). Heating Parylene C to this temperature in the presence of oxygen risked both oxidation as well as severe thermal stress due to the mismatch in thermal expansion properties of Parylene and the PMMA resist. A conductive Cr layer, 15 nm thick, was e-beam deposited; this layer was necessary to dissipate charge during EBL exposure, as the Parylene layer served as an electrical insulator. Following dose characterization (see below), features were patterned at 20 kV with dosage between 240 and 420 μC cm^−2^, and a 30 μm aperture. The Cr layer was removed using CR-7 chromium etchant (Transene Company Inc., Danvers, MA, USA) and the patterned features developed in a solution of methyl isobutyl ketone and isopropanol (1:3) for 18–30 s.

Dose and accelerating voltage were optimized for EBL on Parylene by writing dose test patterns consisting of matrices of squares either 5 or 0.5 μm on a side with dosage varied between 150 and 900 μC cm^−2^, at 10, 15, and 20 kV. Patterns of 5 μm squares were developed and imaged microscopically, and patterns of 0.5 μm squares metalized with titanium liftoff, then imaged by SEM. Size test patterns, squares of diminishing size (5–0.05 μm), were written at 10, 15, and 20 kV with doses of either 420 or 600 μC cm^−2^, then metallized by liftoff and imaged with SEM. A dose test (5 μm square matrix, 75–450 μC cm^−2^) was also performed on a Parylene sample without resist to examine the effect of direct EB radiation on Parylene.

### Device fabrication and characterization

**Figure 2:**
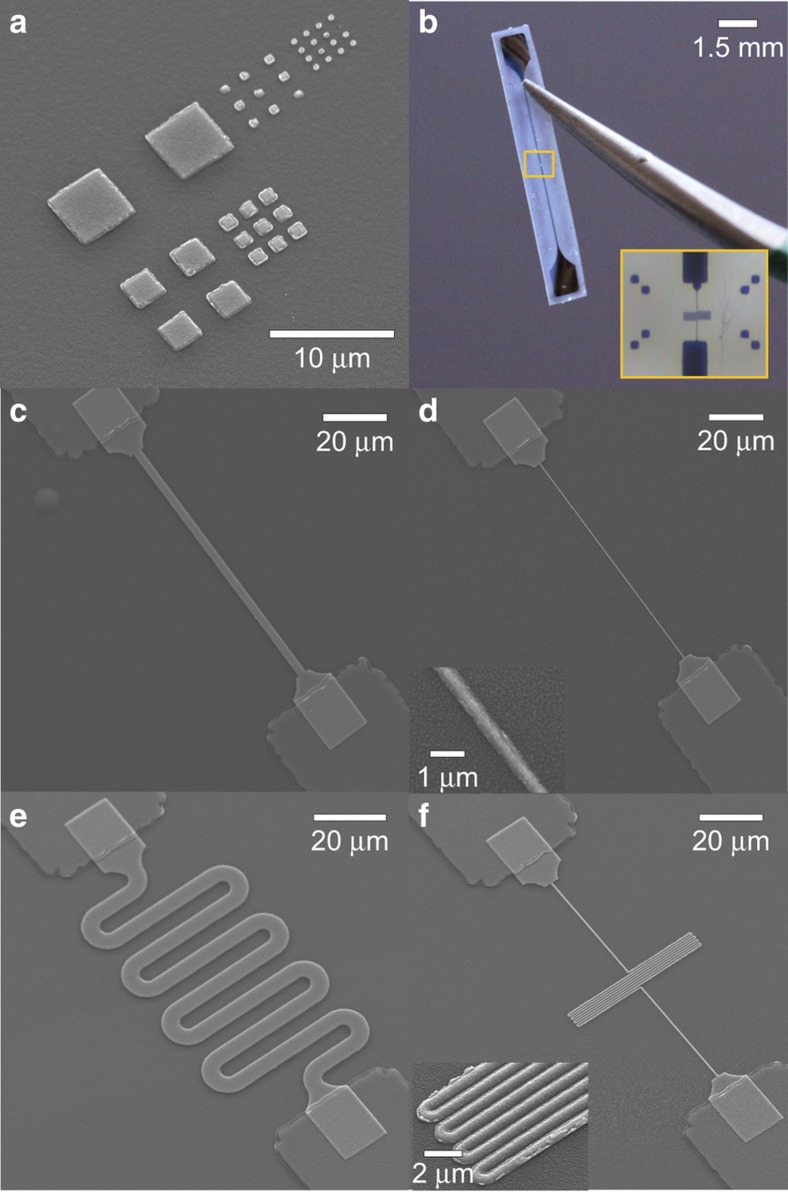
Micrographs of submicron metal structures pattern on Parylene C. (**a**) Squares of decreasing size (5–0.25 μm) patterned to determine the smallest resolvable feature. (**b**) Released Parylene film with encapsulated submicron structure spanning larger metal traces; Straight 0.25 μm Ti trace. (**c**) Straight 2.5 μm Ti trace. (**d**) Serpentine 0.4 μm trace (0.4 μm spacing). (**e**) Serpentine 5 μm trace (5 μm spacing). (**f**) Serpentine 0.4 μm trace (0.4 μm spacing). Parylene C, poly(chloro-p-xylylene); SEM, scanning electron microscope.

Test devices were fabricated to characterize the electrical and mechanical properties of submicron metal structures in free-film Parylene. The devices (10.8 mm *L*×1.75 mm *W*×20 μm *T*) consist of a simple linear circuit patterned in thin-film metal on a Parylene C film, encapsulated in a second layer of Parylene. Two contact pads are connected by a 50 μm wide trace with a 100 μm gap in the center; EBL patterned traces of varied geometry and width were fabricated within this gap to complete the circuit. Figure 2b shows an image of a complete test device. A detailed description of the fabrication is provided in the Supplementary Information section, along with specific parameters for each individual process. Information on the micromachining of Parylene C, and the deposition and patterning of metal films using UV lithography onto Parylene C substrates can also be found in prior publications in further detail^30–32^. Here we provide a brief description of the fabrication of the test devices. First, silicon dyes were coated in 10 μm films of Parylene C to create the base substrate. Titanium structures including contact pads, large electrical traces, and alignment marks were fabricated on top of the Parylene substrate from titanium (150 nm thick). The features were first patterned in a photoresist mask using UV lithography, then metallized with an e-beam deposition of titanium, followed by liftoff in acetone. Then, linear and serpentine traces (widths and spacing: 5; 2.5; 1; 0.7; 0.4; and 0.25 μm) were patterned with EBL (write field 200 μm×200 μm) using the technique described in the above subsection. For all EBL-patterned traces, the contact area with the underlying larger titanium trace was kept a constant 20 μm×15 μm, to both reduce contact resistance and variation in contact resistance. EBL patterns were metallized with titanium (150 nm thick) through e-beam deposition followed by liftoff in acetone. In some experiments, devices were metallized with gold and platinum (150 nm thick) instead of titanium. For all devices the metal structures were subsequently encapsulated in a second layer of Parylene (10 μm), using the same CVD process. Contact pads were exposed with an O_2_ plasma deep reactive ion etch (DRIE), masked by a thick photoresist layer patterned with UV lithography. A second DRIE step followed to etch the device outline. Finally, devices were peeled off the silicon substrate using tweezers.

Two-point direct current (DC) resistance of test devices was measured using contact probes prior to release. Measurements were taken for five devices of each design variation (that is, serpentine or linear submicron traces of varied width and spacings). Following release from the silicon carrier, electrical connection was achieved by adhering wires to the pads with conductive epoxy (silver epoxy, MG Chemicals, Surrey, CA, USA), followed by a two-part adhesive to improve the bond strength. Devices were bent and torqued manually to a bend/torsion angle of at least 90°, while monitoring for device failure, and DC resistance was recorded following deformation. In a second set of experiments, one representative device of each design was flexed using a moving stage and stepper motor, allowing resistance change to be monitored as a function of bend angle (Supplementary Figure SIa). In a final experiment, a single device (0.7 μm wide straight trace) was impinged by a set of calipers, while resistance was monitored, to determine an approximate bend radius at which catastrophic failure occurs.

A second set of devices were fabricated to examine conformality of Parylene insulation around small and densely patterned features. Titanium wires were machined on Parylene through EBL (widths and spacing: 5; 2; 1; 0.75; and 0.4 μm) and coated in CVD Parylene (10 μm thick). UV lithography and O_2_ DRIE was used to cut-out Parylene sections, bisecting the wires. By operating the DRIE with an RF voltage above 100 V, exposed titanium was sputtered away, leaving cleaved cross-sections that were then imaged under SEM.

### Neural microprobe fabrication

**Figure 3:**
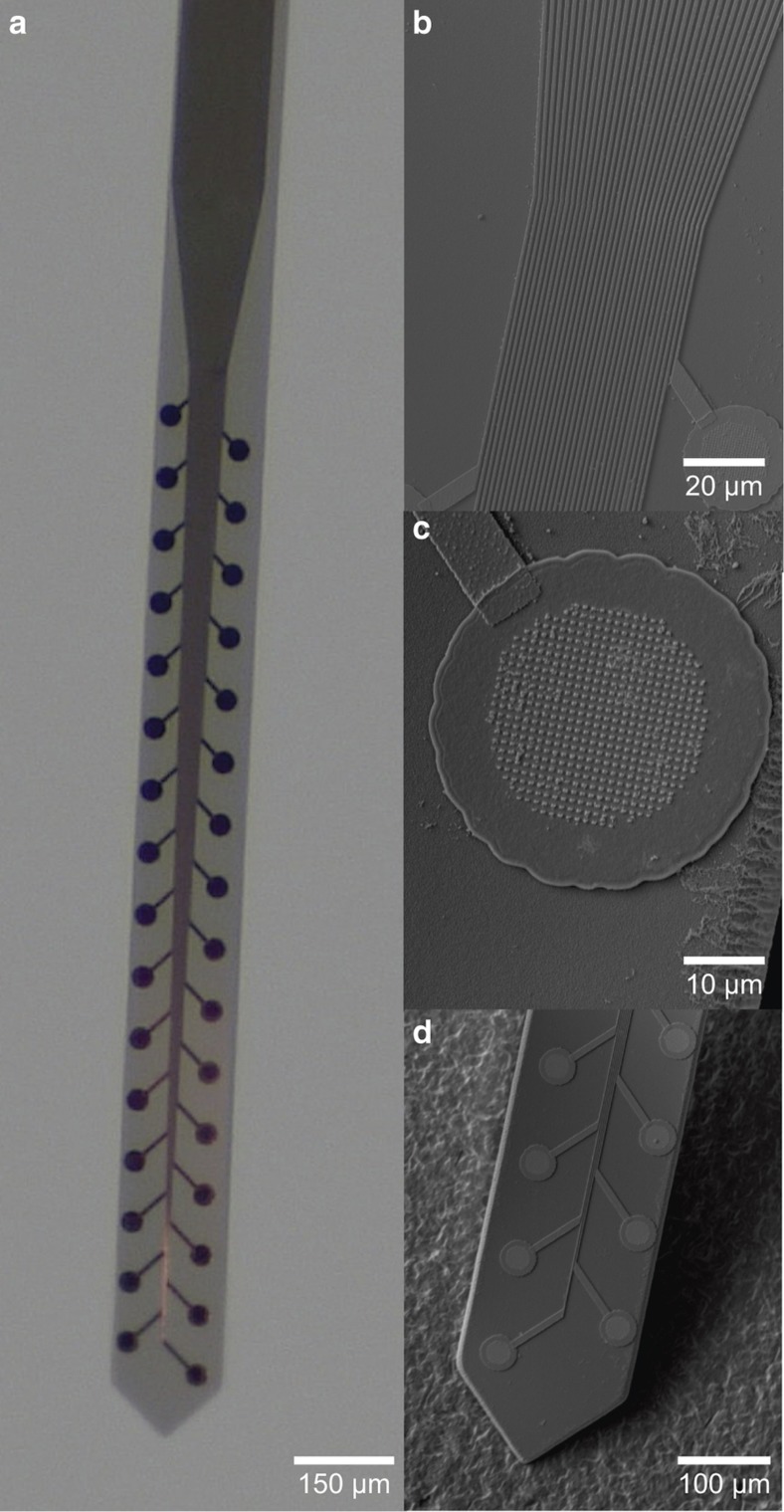
Micrographs of polymer neural microprobe fabricated using polymer micromachining and submicron patterning on Parylene C film. (**a**) Optical micrograph showing entire, fully fabricated probe. (**b**) SEM micrograph showing 32 connective traces with 750 nm pitch and spacing. (**c**) Electrode site with array of submicron patterned ‘bumps’ to increase surface area. (**d**) Close-up image of tapered probe tip. Parylene C, poly(chloro-p-xylylene); SEM, scanning electron microscope.

A polymer microprobe prototype (Figure 3) was fabricated from Parylene and titanium using a derivation of the above techniques. The design consisted of a tapered Parylene shank (2 mm *L*×150 μm *W*×20 μm *T*) supporting a central vein of connective traces branching outwards to circular electrodes spanning either edge of the shank. Electrode recording sites (30 μm diameter) were machined first on the 10 μm thick Parylene base layer using UV lithography, followed by EBL to define the connective traces (750 nm pitch and spacing) and an array of raised ‘bumps’ (400 nm pitch and spacing, Figure 3c) on the center of each electrode site. Following Parylene encapsulation (10 μm), DRIE was used to expose the electrodes and cut-out the probe shape.

## Results

### Challenges of EBL on Parylene

**Figure 4:**
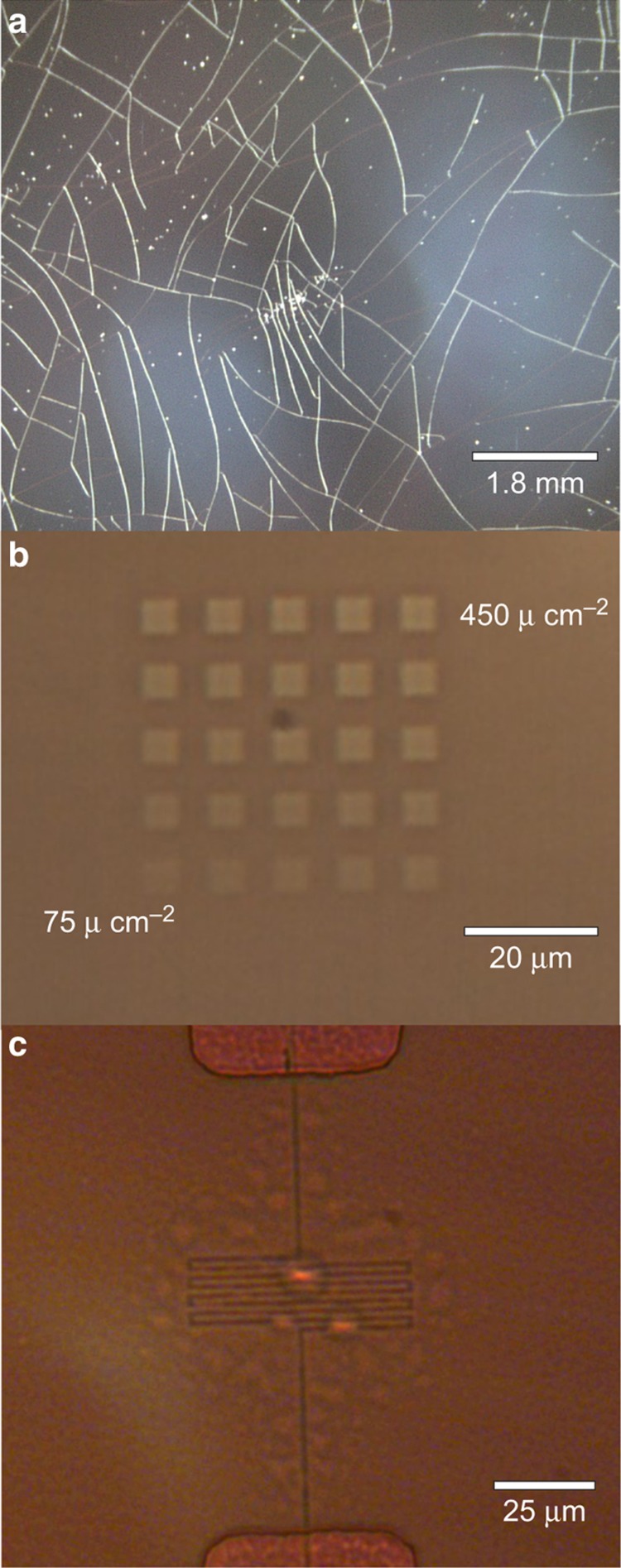
Evidence of damage to Parylene C film during electron beam lithography. (**a**) Cracking of a Parylene film due to thermal stress following preparation of PMMA resist mask. (**b**) Patterns written directly into Parylene film by electron beam at 10 kV and varied doses, highlighting damage induced by electron radiation. (**c**) EBL patterned serpentine trace in PMMA on Parylene, with visible bubbling in the Parylene film due to combination of high exposure dose and dense patterning. Parylene C, poly(chloro-p-xylylene); PMMA, poly(methyl methacrylate); SEM, scanning electron microscope.

The application of EBL to Parylene C films requires solving several critical difficulties. When baking PMMA resist at recommended temperatures (>170 °C), severe cracking of the polymer film was observed (Figure 4a), likely due to thermally induced stress. Lower temperatures (120 °C) and extended bake times (20 min) were substituted with success, but even this comparably low temperature exceeds the glass transition temperature of Parylene C^33^, potentially changing the material and mechanical properties of the film.

Electron beam exposure of bare Parylene C films generated significant charging, as would be expected of an insulating substrate. The addition of a metal overcoat 10–20 nm thick eliminated the charging, allowing for beam focusing, without impeding EBL patterning. Chromium was selected due to its strong adhesion to Parylene, ease of removal through chemical etching, and low atomic mass, which facilitated imaging pre-patterned metal structures for alignment. Direct exposure of Parylene to the electron beam induced visible damage (Figure 4b) to the film, though we were not able to distinguish whether the damage was thermal or direct ablation of the Parylene. Regardless, this is indicative of significant absorption of the beam, and suggests that during lithography there may be considerably less electron backscattering compared to silicon substrates. Similar damage was not observed on Parylene films prepared with EBL resist as described in the Materials and Methods section, likely because the majority of the energy was absorbed by the PMMA bilayer; however, the presence of bubbles (Figure 4c) under the Parylene film was observed in examples of high-density patterning, suggesting localized heating in areas with dense EBL patterning.

### Fabrication results

Under optimized exposure conditions (20 kV, 240–420 μC cm^−2^), features as small as 250 nm could be patterned and metallized without damage to the underlying Parylene film or distortion of the structure. Features patterned with higher doses appeared over exposed, with rounded corners and broadened dimensions, and in some cases damage to the Parylene film was observed. Features patterned with lower doses did not resolve fully after development, and metallization of complete structures failed. Similarly, patterning with lower accelerating voltage yielded discontinuous films following metallization. As with typical EBL, lower dosage was required for dense patterns (for example, serpentine traces with spacing<750 nm) as the proximity effect lead to over exposed and collapsed structures. Features smaller than 250 nm did not resolve during size testing, which we believe to be a limitation on our development chemistry and not the patterning tool. For a resist thickness of 570 nm, this bounds the aspect-ratio achievable with this approach below 4:1. Thinner resist masks should allow for patterning of smaller structures (<250 nm), but would necessitate reducing the thickness of the resulting metal structure so that clean liftoff can still be achieved. Even with the current resist mask, liftoff residue was sometimes observed on the sidewalls of smaller structures (Figure 2f, inset), possibly a consequence of thick metal (150 nm) relative to the resist layer thickness (400 nm). Metallization was achieved with gold and platinum in addition to titanium (all 150 nm thick), however, gold adhesion to bare Parylene was unacceptably poor. Adhesion between platinum or titanium and the Parylene film was very strong, but the high heat required for the platinum deposition induced stress in the metal films due to mismatch of thermal coefficient of expansion, and occasionally caused cracking in the larger structures. Though no cracking was observed in the smaller (<5 μm) structures patterned with EBL, titanium was selected for the metal in all experimental devices to avoid film stress as a confounding factor in analysis.

**Figure 5:**
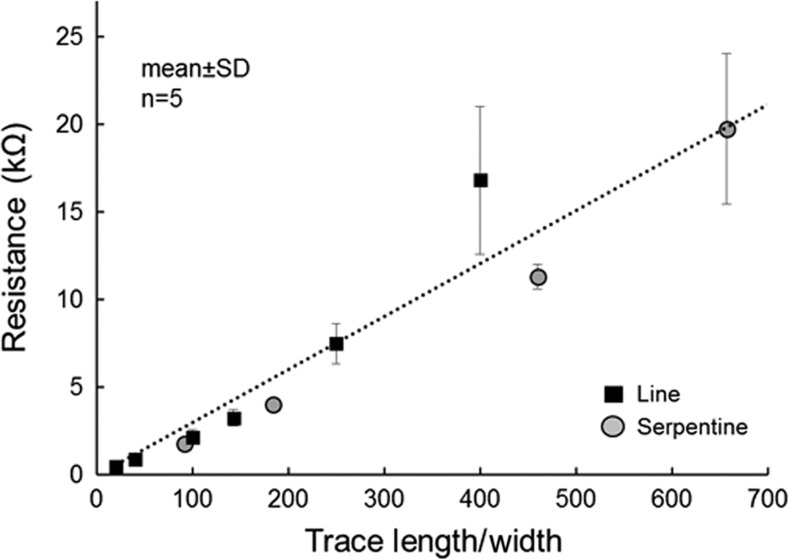
Plot of resistance vs. length-to-width ratio of patterned test structures: straight (square) and serpentine (circle) traces. Each data point represents the mean of measurements from five separate devices, and error bars depict,±one standard deviation. DC resistance was measured using two-point contact with the devices in a flat orientation. Resistance contribution from contact pads and larger traces has been manually subtracted. DC, direct current.

Conducting traces with widths as small as 250 nm and trace-to-trace spacing as low as 400 nm were successfully and repeatedly fabricated. Figure 2 displays SEM micrographs of representative devices prior to encapsulation. Under DC characterization, traces were Ohmic, and resistance values (plotted in Figure 5) did not change measurably following repeated manual folding, bending and torsion of the devices. The combined DC resistance of the contact pads and traces that establish connections to the EBL patterned structures was measured directly by 2-contact probe as 2.1 kΩ. In Figure 5, this contribution has been subtracted from each data point, to present the resistance of solely the EBL patterned structures. Resistivity of the thin-film titanium in EBL patterned structures was calculated as 3.5–6.4 μΩ m using the standard expression for a wire (*ρ*=*RLτ*/*W*), where *τ* is thickness of the deposited titanium (150 nm) and length (*L*) and width (*W*) were experimentally varied. This is an order of magnitude higher than bulk values for titanium (0.42 μΩ m), and higher than the resistivity of unpatterned titanium films evaporated to the same thickness (1–2 μΩ m, consistent with prior reports)^34^. The increase in resistivity may indicate contributions from grain size or poor film continuity negligible in large films, which could become measurable at submicron widths. Although higher impedance is undesired, the measured resistivity values are not high enough to impede use in devices. Contact resistance between the EBL defined structures and larger contact pads and traces was negligible (<50 Ω). In practice, attaining this low contact resistance required matching the metal thickness of the two structures, achieving sidewall profiles without undesired ‘wing-tipped’ residues following liftoff, and using comparatively large (20 μm×15 μm, visible in Figures 2c–f) area contacts. In devices fabricated without these considerations, contact resistances were unacceptably high, and such devices often failed at the contact region entirely under even mild strain.

Little to no change in resistance was observed following manual bending and torsion (<1% Δ*R*), and similar results were recorded for those devices bent by motorized stage, while recording resistance. Supplementary Figure SIb shows representative data for a single device from this set of experiments. On the basis of these results, the EBL patterned traces were shown to be robust to a bend angle of at least 0.9 mm. For a single tested device, resistance was steady until reaching a bend radius of between 200 and 300 μm, at which point catastrophic failure occurred.

**Figure 6:**
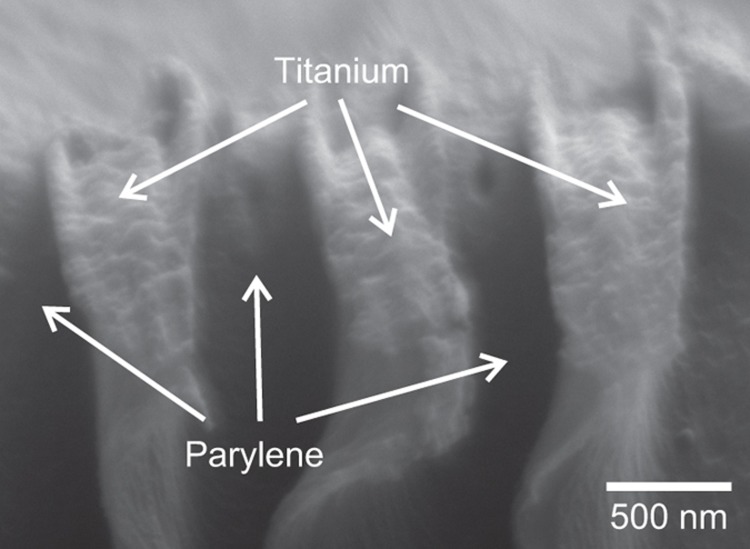
SEM micrograph showing the bisected cross-section of titanium wires (400 nm widths and spacing) encapsulated in Parylene C. Despite close spacing of the wires, intercalary insulation of Parylene C is clearly visible. Parylene C, poly(chloro-p-xylylene); SEM, scanning electron microscope.

Figure 6 shows a representative SEM image of the cross-section of a device bisected with DRIE, exposing EBL-patterned titanium wires encapsulated in Parylene film. Despite the small pitch and spacing (400 nm) of the wires, the intercalary space between them is filled with conformal Parylene C. Similar observations were recorded for larger sets of wires with varied spacing. The conformality of the vapor deposited Parylene appears excellent, even around structures patterned with the densest achievable spacing.

The 32-site neural microprobe is shown in detail in Figure 3. Titanium traces up to 2 mm long, with 750 nm pitch and width, were successfully patterned and encapsulated in Parylene. No evidence of trace collapse or cracking was observed despite the flexibility of the probe’s Parylene base. Line resistance for the traces on the prototype probe was not measured, though it can be extrapolated as 150 kΩ from measurements of test structures. In a functional device titanium would be replaced, likely with platinum, and relevant impedance values would be recorded at 1 kHz instead of DC, so this estimate has utility only as an upper bound on expected line resistance. Raised structures 150 nm tall and 400 nm across were patterned throughout the center of each electrode site to increase the effective surface area, as a means to improve charge storage capacity and lower electrode impedance. The effective surface area was calculated as 40% larger than the geometric surface area.

## Discussion

The results of this study bode well for the development of new flexible and biocompatible microdevices with metallic features with submicron resolution. Although Parylene is poorly suited for EBL, with proper preparation of the substrate resolution as low as 250 nm was achieved, and smaller structures still may be patterned on Parylene with a different resist mask (either choice of resist or change in thickness) or developer chemistry. Submicron structures, including electrical connections, retained good adhesion and encapsulation despite repeated and severe manipulation. The vapor deposited insulation was incredibly conformal, despite the small size and tight spacing of structures. No breaks in electrical traces were observed during manual flexion or torsion, suggesting devices are robust despite nm size scale. The bend radius at which these traces fail could not be determined accurately, in part because the submicron traces could not be imaged at high enough optical magnification to determine whether breaks measured at very low bend radii (200–300 μm) occurred in the submicron traces. Prior work has shown that thin-film platinum traces fabricated on Parylene retain integrity down to bend radii as low as 100 μm, and under fatigue testing up to 100 000 bends^35^. In that work, traces had widths and lengths on the order of a millimeter, and failure occurred due to propagation of micro-cracks across the entire span of the trace; given the small width of traces tested here, failure under fatigue testing might occur in fewer bends.

The reduction in feature size offers greatly expanded capabilities for development of new technologies, including greater feature density, and reduced footprint. The prototype neural probe shown here is an example of how improved fabrication capabilities can aid polymer MEMS development for biomedical devices. At 32 recording sites on a single polymer shank (133 per mm^2^), this device has more than double the electrode density of any prior polymer probe^9^, and four times the density of any prior Parylene C-based probe^36^. This is primarily achieved through the narrow width and small spacing of the connective traces, although patterning of the recording sites is designed to increase surface area and reduce electrochemical impedance, allowing for reduction in electrode area without sacrificing fidelity. Further reductions in trace width and spacing are possible, as demonstrated by success patterning traces with 250 nm width and 400 nm spacing. Although line resistance and parasitic capacitance will grow with diminishing trace width and spacing, silicon devices have successfully employed EBL to produce 200 nm lines and spacing for neural probes that contribute marginally to total electrode impedance at typical (1 kHz) operating frequencies^37^. Similar high-density patterning is in principle achievable for Parylene microprobes. Removing the limitation on recording density opens the door to a new generation of polymer neural probes, with mechanical and material properties better suited for chronic implantation than silicon.

The above is but a demonstrative example of how this method may be applied. Potential applications not explored here include free-film devices with electrical components, such as resistors and capacitors, directed written into the film using dense serpentine traces and interdigitated comb designs, respectively, or high-density retinal electrodes, with large arrays of stimulating electrodes small enough to stimulate single photoreceptors. Improvements in resolution, enabling nanoscale structures, should be possible with EBL on Parylene using only minor modifications of the described method. By using a thinner resist bilayer, higher aspect-ratio structures with smaller critical dimensions could be fabricated, however, this may necessitate depositing thinner metal layers to achieve liftoff successfully. Alternative methods, including the use of high (for example, 100 kV) e-beam energies, may not be feasible due to the damage incurred by Parylene films from higher-energy exposure. Reducing feature size could enable flexible, biocompatible structures with devices that exploit nanoscale phenomena such as localized surface plasmon resonance for biochemical sensing. Although this work focused on patterning thin-film structures within Parylene encapsulation, the method is trivially modified for direct patterning or modification of Parylene surfaces as well.

Ultimately, batch-scale fabrication methods must be developed to produce such devices in large numbers and lower cost. The EBL used here is inherently slow and serial, with exposures for a single neural microprobe taking up to 2 h, and cannot easily be adapted to wafer-scale processing. For experimental development and design, or for projects that require the production of only a handful of devices, the time constraint is marginal compared with the time required for steps such as packaging. For devices intended for commercialization, however, EBL is likely too arduous and cost prohibitive. Future work will require large-area pattern transfer methods, to parallelize fabrication following development and testing using the technique presented here.
