# Supplementary figures and images for: Electron-beam lithography for polymer bioMEMS with submicron features
Source: Microsyst Nanoeng. 2016 Nov 7;2:16053. doi: 10.1038/micronano.2016.53 (PMC6444738; doi:10.1038/micronano.2016.53)

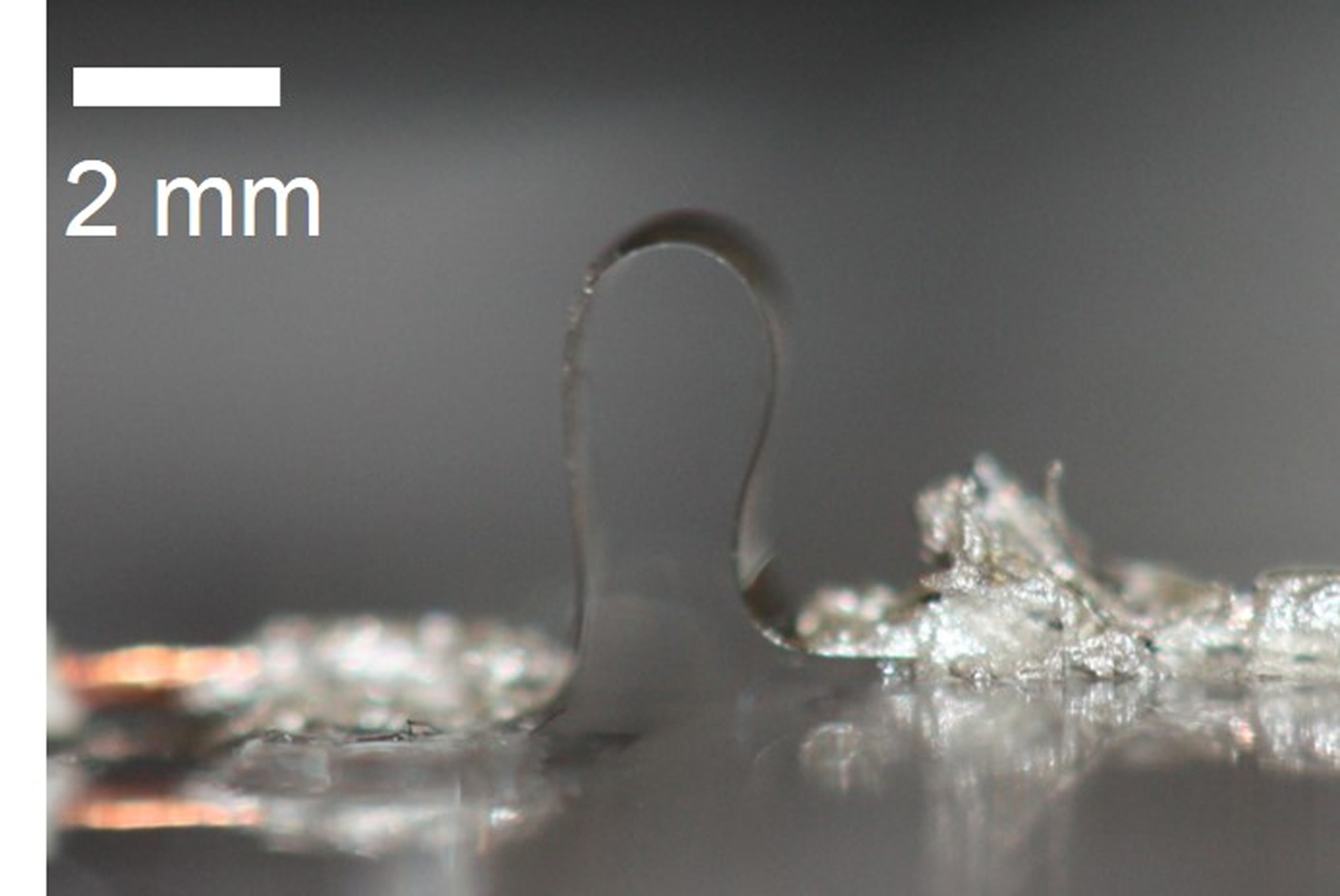

Supplement: Supplementary Figure 1 [file micronano201653-s1.tif]

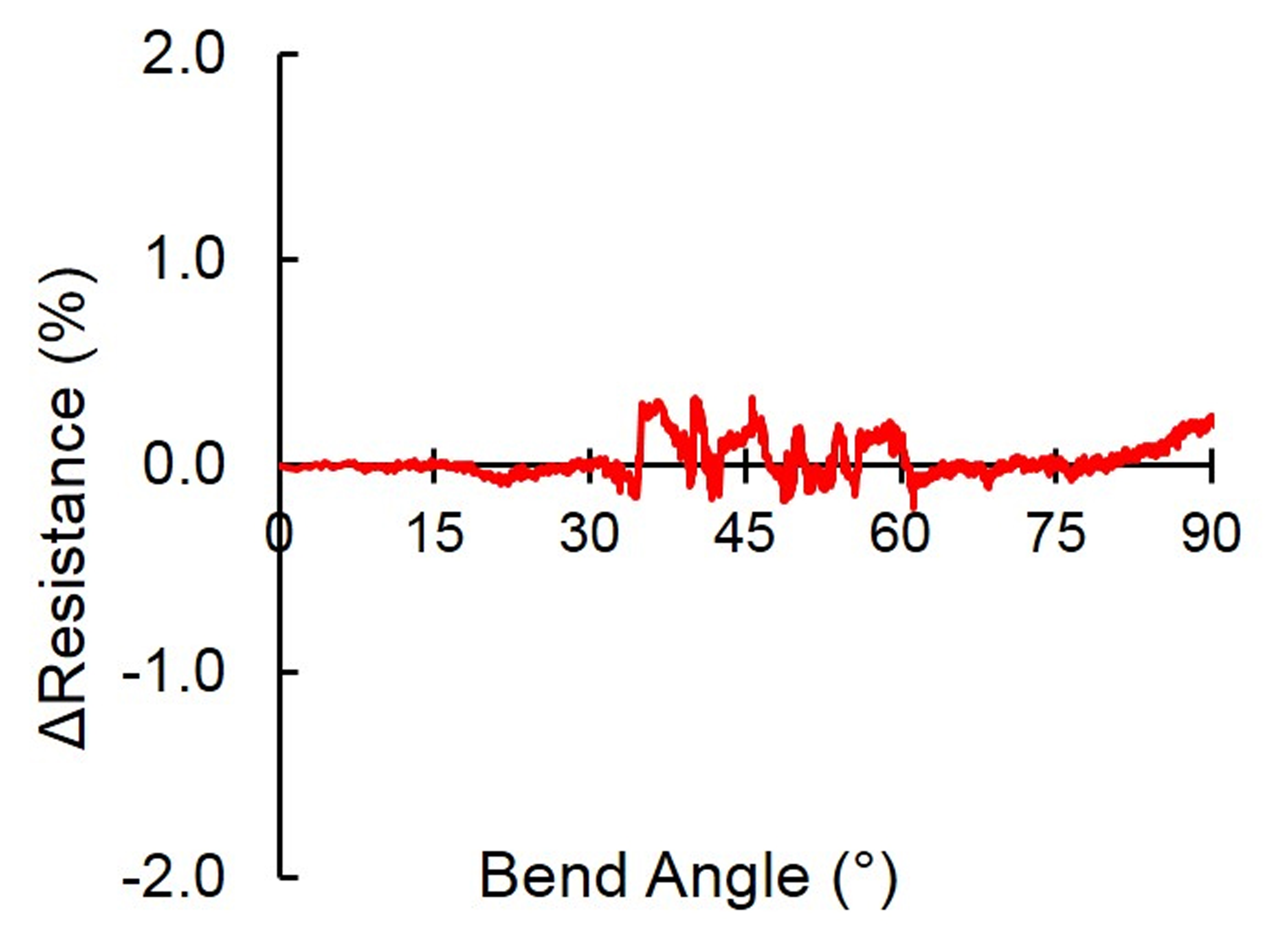

Supplement: Supplementary Figure 2 [file micronano201653-s2.tif]
