# Supplementary material for: Electron-beam lithography for polymer bioMEMS with submicron features
Source: Microsyst Nanoeng. 2016 Nov 7;2:16053. doi: 10.1038/micronano.2016.53 (PMC6444738; doi:10.1038/micronano.2016.53)
Supplement: Supplementary Information [file micronano201653-s3.pdf]

Supplementary file

Supplemental Information for Electron-beam lithography for polymer bioMEMS with submicron features

Kee Scholten and Ellis Meng

Microsystems & Nanoengineering (2016) **2,** 16053; doi:10.1038/micronano.2016.53; Published online: 7 November 2016

DETAILED FABRICATION

A detailed description of the fabrication process used in this study is presented here. Though several devices are presented in this paper, all were fabricated using the generalized process described below.

Devices consisted of a base Parylene substrate (10 μm thick), an initial metal layer defined using UV lithography and lift-off (150 nm), a second metal layer defined using electron beam lithography (EBL) on polymer and lift-off (150 nm) and an encapsulating top layer of Parylene (10 μm thick).

Silicon dies (35 mm × 35 mm) were used as carrier substrates to support the Parylene C films. Dies were chosen over full wafers due to the limited size usable in the electron beam lithography system we had available. Dies were prepared from 4 inch prime Si wafers using a hand scribe. Dies were baked in an oven at 110 °C overnight to remove any residual moisture, then coated in a 10 μm conformal layer of Parylene C at room temperature using a commercial Parylene CVD system ((PDS 2010 Labcoter, Specialty Coating Systems, Indianapolis, IN). Parylene thickness was confirmed using profilometry.

The initial metal layer comprised alignment marks, contact pads, electrode sites, and other large (> 10 μm minimum dimension) structures. In all experiments and figures presented in the publication, the metal features are made from titanium. Both gold and platinum were tested, however the gold had poor adhesion to the Parylene and the heat generated during deposition of platinum created undesired thermal stress. These issues were not present with titanium and so all subsequent work used titanium exclusively. Features were defined in a photoresist mask (AZ 5214, 2 μm thick) using standard UV lithography. The steps were as follows: (1) after spinning the resist layer to the desired thickness, (2) dies were baked on a hotplate at 90 °C for 70 s (3) exposed to UV light at 37.5 mJ cm^{-2} through a negative chromium mask (4) baked at 110 °C for 40 s to initiate the image reversal (5) flood exposed without mask to UV light at 150 mJ cm^{-2}, and finally (6) developed in a 1:4 solution of AZ340 developer and deionized water.

Titanium was deposited on dies with an electron beam physical vapor deposition tool (Temescal BJD-1800) at a rate of 1.2 Å s^{-1}; to minimize thermal damage or stress in the Parylene film, deposition was broken into 50 nm increments, with 15 min pauses between each deposition. Following deposition, the photoresist mask was dissolved in subsequent baths of warm acetone (10 min, ~ 60 °C) and room temperature acetone (10 min), followed by rinsing in isopropanol and deionized water.

The second metal layer comprised features patterned using electron-beam lithography and included linear and serpentine traces for test devices, and the connective traces and 'bump' structures for the prototype neural probe, all < 5 μm in minimum dimension. Features were defined in a PMMA bilayer mask using a modified EBL process. PMMA 495k (6% solids in anisole) was spun to 400 nm thickness and baked on a 120 °C hotplate for 20 min to remove residual solvent, followed by PMMA 950k (2% solids in anisole) spun to 170 nm thickness and baked at 120 °C for an additional 20 min. A 15 nm thick chromium layer was then deposited using e-beam PVD at a rate of 1.4 Å s^{-1}. A Raith 150 e-beam lithogrpahy tool was used to pattern features at exposures between 10–20 kV and 75–600 μC cm^{-2}. A 30 μm aperture and 200 μm × 200 μm write field was used for all fabrication. Patterns were aligned using titanium alignment marks defined in the initial metal layer. The higher atomic mass titanium layer was easily visible through the PMMA and chromium layers under SEM owing to the higher degree of electron backscattering.

Following exposure, the chromium layer was removed with immersion in a bath of CR-7 chromium etchant (Transene Company Inc., Danvers, MA) for approximately 15 s. The patterned features were developed in a solution of methyl isobutyl ketone and isopropanol (1:3) for 18–30 s, followed by a 30 s bath in isopropanol to rinse off residual developer. The second titanium layer was deposited using ebeam PVD (150 nm thick) in the same manner as above, and the PMMA mask dissolved during lift-off using subsequent baths of warm acetone (60 °C) and room temperature acetone for 10 min each, followed by rinses in isopropanol and deionized water.

The encapsulating top layer of Parylene was deposited 10 μm thick using CVD with the same commercial system. Two etch steps were performed to expose the contact pads and to cut-out (release) the individual devices. A photoresist mask (AZ 4620) was prepared 15 μm thick and patterned using UV lithography. The process was as follows: (1) after spinning the resist layer to the desired thickness (2) dies were baked on a hotplate at 90 °C for 360 s, (3) exposed to UV light at 550 mJ cm^{-2} through a positive chromium mask (4) developed in a 1:4 solution of AZ340 developed and deionized water and (5) hard-baked on a hotplate at 90 °C for 10 min followed by 15 min in an oven at 90 °C under vacuum.

Deep reactive ion etch (DRIE) was performed to a depth of 10 μm using an Oxford PlasmaLab 100 tool. Dies were first mounted to 4 inch Si carrier wafers using a thin layer of photoresist, and etched using a modified Bosch process with an O_2 plasma etchant and C_4F_8 passivation layer. 120 cycles were required to etch a depth of 10 μm, with an inductively coupled plasma power of 700 W and RF power of 10 W (< 100 V) for both etch and passivation. Temperature of the devices was not monitored or controlled, though a helium backstream was used to cool the dies. Following the first DRIE etch step, the photoresist

Department of Biomedical Engineering, University of Southern California, Los Angeles, CA 90089-1111, USA.
Correspondence: Ellis Meng (ellis.meng@usc.edu)

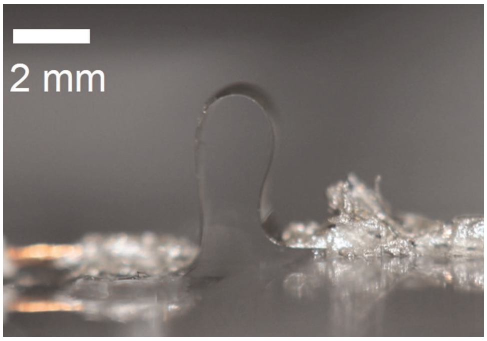

Figure S1 Side image of a test device (700 nm serpentine trace) bent using a movable stage, while DC resistance is recorded.

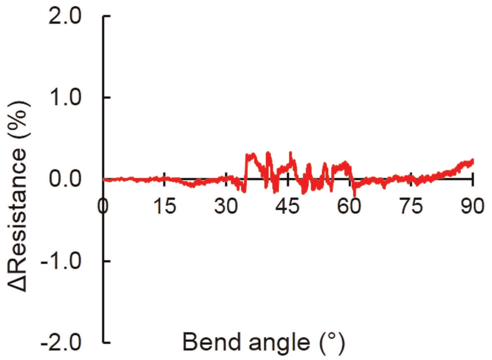

Figure S2 Representative data from mechanical characterization (700 nm serpentine trace): fractional change in resistance plotted against bend angle.

mask was stripped using baths of acetone and isopropanol. A second photoresist mask was deposited and patterned in the same manner, and used to mask a second DRIE step which cut-out the individual devices. The final photoresist mask was stripped, and individual devices were released by placing droplets of water at corner and peeling the devices off the wafer using tweezers. The water droplets spread between the Parylene and silicon, assisting in release.
